# Reconstructing Three‐Dimensional Optical Anisotropy with Tomographic Müller‐Polarimetric Microscopy

**DOI:** 10.1002/advs.202502075

**Published:** 2025-05-08

**Authors:** Yang Chen, Arthur Baroni, Torne Tänzer, Leonard Nielsen, Marianne Liebi

**Affiliations:** ^1^ Center for Photon Science Paul Scherrer Institut Villigen PSI 5232 Switzerland; ^2^ Institute of Materials Ecole Polytechnique Fédérale de Lausanne (EPFL) Lausanne 1015 Switzerland; ^3^ Department of Physics Chalmers University of Technology Gothenburg 41296 Sweden; ^4^ School of Computing, Engineering and Physical Sciences University of the West of Scotland Paisley PA1 2BE UK

**Keywords:** 3D ultrastructure, bio‐imaging, human trabecular bone, reconstruction, tomographic polarized light microscopy

## Abstract

Most visible light imaging methods using polarization to obtain ultrastructure information are limited to 2D analysis or require demanding phase measurements to be extended to 3D. A novel 3D polarized light imaging technique based on Müller‐matrix formulations is introduced which numerically reconstructs 3D optical birefringence, that is anisotropic refractive indices and optical axis orientation, in each volumetric unit of sample. The new method is demonstrated, tomographic Müller‐polarimetric microscopy, in simulation and using experimental data of 3D macroscopic sample of human trabecular bone sample, where the local main orientation of nanoscale collagen fibers is extracted with a resolution of ≈ 20 µm. Tomographic Müller‐polarimetric microscopy offers a low‐cost and experimentally simple imaging approach to access the ultrastructure which is not directly resolvable, in a wide range of biological and composite materials.

## Graphical abstract

Tomographic Müller polarimetric microscopy is a novel imaging technique that resolves 3D birefringent properties of bulky samples, unveiling hierarchical nanostructures at microscopic resolution. Based on incoherent visible‐light polarimetry, it achieves experimental simplicity by eliminating phase measurements. The method is demonstrated on a human trabecular bone sample, extracting local collagen fiber orientations with a resolution of ≈20 µm.

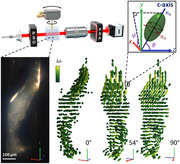

## 1 Introduction

Ultrastructure, a term referring to nanoscale structures too small to be imaged using standard optical light microscopy, can be probed indirectly by measuring their interaction with photons, electrons or neutrons resulting in scattering, diffraction or a change in polarization state. This allows to attain the underlying ultrastructure in an extended field of view, as used in polarized light microscopy since the 19^th^ century.^[^ ^1^ ^]^ Alignment of nanostructure, such as collagen fibers^[^ ^2^, ^3^ ^]^ or nerve fibers in brain^[^ ^4^, ^5^ ^]^ leads to anisotropic scattering/diffraction and refraction, producing birefringence and anisotropic attenuation, i.e. diattenuation/dichroism,^[^ ^6^ ^]^ in the visible light frequencies. The causes are molecular geometry (intrinsic birefringence), and submicron structural order (form birefringence).^[^ ^7^ ^]^ While for qualitative measurements a single exposure of the sample between cross polarizers is enough, quantitative measurements need acquisition at several polarization states, which can be achieved by manipulating a set of polarization elements. The most common and complete method to measure quantitatively polarizing effects is through Müller polarimetry, an intensity‐based direct‐imaging technique where the polarizing properties of a sample are retrieved with a four‐by‐four matrix that defines the anisotropic light‐matter interaction, the Müller matrix.^[^ ^8^ ^]^ The measured polarizance of a sample is a direct result of the in‐plane projection of its dielectric tensor, a physical quantity that describes the refractive index in 3D. Uniaxial materials (most biological tissues) exhibit simply retardance and diattenuation, therefore measuring the anisotropic real and imaginary parts of the projected dielectric tensor are the main aim of many quantitative polarimetric methods, while the isotropic part responsible for optical phase is not measurable in Müller polarimetry.

Polarized light microscopy, has been explored mainly for unveiling the 2D optical anisotropy by imaging thin layers.^[^ ^2^, ^9^, ^10^ ^]^ Pushing from layer probing to 3D analysis, combining Mueller‐matrix‐based polarized light microscopy with digital holography allows depth‐resolved anisotropy and depolarization study of scattering samples.^[^ ^11^, ^12^, ^13^, ^14^, ^15^ ^]^ Recent advances enabled polarized light microscopy to retrieve the out‐of‐plane orientations of brain section,^[^ ^5^, ^16^ ^]^ assuming a constant fiber density. Lately, the dielectric tensor describing the optical anisotropy was retrieved for bulky samples using polarization‐sensitive optical diffraction approaches with Born^[^ ^17^, ^18^ ^]^ and Rytov approximations.^[^ ^19^ ^]^ They perform either 3D tomographic reconstruction with 2D scattered fields,^[^ ^18^, ^19^ ^]^ requiring experimentally demanding phase measurement, or angular illumination scans with a limited range,^[^ ^17^ ^]^ leading to a missing wedge concern.

Here, we report a new method, tomographic Müller‐polarimetric microscopy (TMPM), to reconstruct the 3D birefringence distribution within bulk samples. The polarization‐resolved technique reconstructs parameters based on Müller‐matrix formalism from projections at different angles to rebuild the anisotropic geometry resolved in subvolumes, or voxels in the micrometer scale. The key strategy is to utilize Müller polarimetric measurements to convert the bulk birefringence information into light intensity that can be conveniently captured by commercial cameras or photodetectors, thus avoiding the complicated interferometric measurement in diffraction‐based techniques. Multiple projections of samples are measured by rotating it around two axes within the light path. The polarizing effect of each voxel is modeled by an index ellipsoid featured with a local optical axis and birefringence, resulting in a Müller matrix depending on the projection angles. An optimization algorithm is used to minimize the error between the simulated and measured intensities. The immutable matrix multiplication and their angular dependency leads to a high non‐convexity of this inverse problem. We tackle this by utilizing a Nesterov accelerated gradient descent method^[^ ^20^ ^]^ with rigorous analytical gradient functions to improve convergence accuracy and speed.

We first validate the new technique by reconstructing the 3D anisotropy of a simulated spiral birefringent sample. Then we demonstrate the method on a sample of human trabecular bone of a size of 375 × 820 × 375 µm^3^ (16 × 35 × 16 in voxels), enabling the access of the collagen fiber geometry at a voxel size of ≈20 µm, which we validated by measuring the same sample with already established synchrotron small‐angle X‐ray scattering tensor tomography.^[^ ^21^ ^]^ It resolves the long‐existing concern of using phase‐free polarized light microscopy to measure 3D optical anisotropy, opening a new roadmap to visible‐light 3D structural imaging of high accessibilities and low experimental complexity.

## 2 Results

### 2.1 Principles of Tomographic Müller‐Polarimetric Microscopy

**Figure 1:**
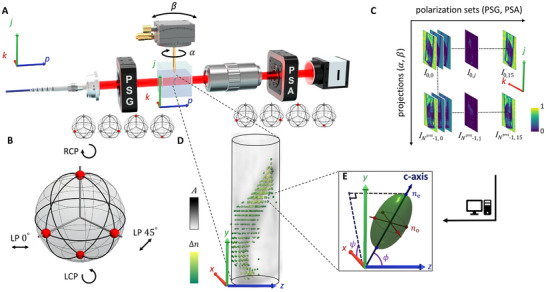
Tomographic Müller‐polarimetric microscopy. A) Schematic of the setup. The light travels through the polarization modulators of PSG, the birefringent sample, and PSA in sequence, and is captured by a charge‐coupled device camera. The tomography is performed on *N* ^proj^ projections by using a goniometer to tilt and rotate the sample at different angles, that is (α, β). B) Polarization states used in PSG and PSA, illustrated on the Poincaré sphere. The four states are composed of the two circular (pole, RCP, and LCP) and the two linear (equator, LP at $0^{\circ }$ and $45^{\circ }$ respectively). C) Projected light intensity images recorded by the camera, resolved by the polarization set (in total 16 in the measurement) and projection set (in total *N* ^proj^ in the measurement). D) Zoom‐in of the sample with a sketched structural distribution, shown as index ellipsoids of birefringence Δ*n* and absorption *A* that are reconstructed using all projections in (C). E) A typical index ellipsoid, characterized with a slanted c‐axis, and the anisotropic refractive indices of e‐ and o‐waves, respectively *n*_e_ and *n*_o_, producing the birefringence Δ*n*  = *n*_e_  − *n*_o_.

3D optical anisotropy in biological tissues is often limited to uniaxial birefringence, commonly modeled as an index ellipsoid. Index ellipsoid is a classical concept deployed to describe crystal birefringence,^[^ ^22^ ^]^ with an axis where no double refraction occurs, known as optical axis (denoted as *c‐axis*). A uniaxial index ellipsoid is specified with the polar and azimuthal angles (ϕ, ψ) orienting its c‐axis, and the non‐degenerate refractive indices *n*_o_ and *n*_e_, respectively of the ordinary (o‐wave) and extraordinary waves (e‐wave), as sketched in **Figure** **1**E. To measure the 2D projection of the 3D anisotropy (see Equation 1 in Experimental Section and Methods for the incidence‐dependent e‐wave index *n*_E_), the Müller polarimetry is employed, schematically depicted in Figure 1A (further detailed in Experimental Section and Methods, and shown with more visual information in Note S1; Figure S1, Supporting Information). A set of four independent polarization states are generated and analyzed by the polarization state generator (PSG) and analyzer (PSA), respectively, to measure the polarization features. In the measurement, the four states used are right‐circular polarization (RCP), left‐circular polarization (LCP), and linear polarization (LP) at $0^{\circ }$ and $45^{\circ }$, as illustrated by the four red points spread on the Poincaré sphere in Figure 1B, which are then varied separately in both PSG and PSA as illustrated in Figure 1A, resulting in 4 × 4, totally 16, polarization sets and measured images (Figure 1C). An index‐matching liquid, here an immersion oil with a close refractive index to that of the sample material, is used to cancel as much as possible the unwanted refractions generated at the edge of the sample for microscopic illuminations.

Conventional 2D polarized light microscopy retrieve only the cumulative optical properties along the beam path, focusing usually on the total linear retardance δ^tot^ and its projected fast‐axis orientation θ^tot^. TMPM reconstructs the full 3D anisotropies in each voxel in the sample‐fixed coordinate system (*x*, *y*, *z*), sketched with index‐ellipsoid distributions as exemplified in Figure 1D, with tomographic projections of the sample around two axes, at the rotation and tilt angles (α, β) within the laboratory coordinate system (*k*, *j*, *p*), where *p* is the light ray direction. The use of two tomographic axes was introduced for scattering tomography in the X‐ray regime, the reference method used here namely small angle X‐ray scattering tensor tomography (SAXSTT), as a way to access 3D orientations of local structures.^[^ ^21^ ^]^

### 2.2 Forward Model and Reconstruction Strategy

To simplify the light‐propagation ray‐tracing model, only parallel straight ray of light, that is ballistic light, where only one ray hits each pixel of the detector, is assumed (see Experimental Section and Methods for more discussion). This assumption works in the condition of small variation of the o‐wave index *n*_o_^[^ ^23^, ^24^ ^]^ and low birefringence^[^ ^23^, ^25^, ^26^, ^27^ ^]^ (realistic for biological samples, e.g., birefringence has been reported with a typical magnitude of ≈10^−4^‐10^−3^ in collagen,^[^ ^3^, ^28^ ^]^ brain fibers,^[^ ^16^ ^]^ muscle fibers,^[^ ^29^ ^]^ carcinoma,^[^ ^14^ ^]^ retina,^[^ ^30^ ^]^ and hair^[^ ^31^ ^]^), allowing to neglect the refraction and double refraction of light. For each projection corresponding to tomographic angles (α, β) we have access to the synthetic Müller matrix *M*_(α,β)_ of the sample at each pixel (*k*, *j*) of the sensor, integrated through the depth along the *p* axis, by using the related 16 polarization‐resolved projection images. For each voxel in the path, the polarizing effects are modeled as a uniaxial index ellipsoid specified by (*n*_e_,ϕ, ψ) and expressed as a Müller matrix depending on these parameters and the incidence angle (α, β) of the probing light (see Equations 1 and 7 of Experimental Section and Methods for the calculus, and Note S2, Supporting Information for more details). The measured Müller matrix *M*_(α,β)_ can be considered as the product of the projection‐dependent Müller matrices of all voxels in the light path (see Experimental Section and Methods Equation 8).

Since the forward model links the measured intensities to the index ellipsoid at each voxel, we can reconstruct the latter using an optimization approach over the mean‐squared‐error ε^I^ optimization between the measured intensities *I* and the computed intensities $\hat{I}$ (Equation 10 of Experimental Section and Methods). As this inverse problem, based on Müller polarimetry, is highly nonlinear and non‐convex, due to the multiplication of non‐Abelian Müller matrices, additional regularization constraints based on prior knowledge and assumptions about the system must be imposed in order to reliably obtain convergence. As most of biological samples for those structural studies display continuous variations, we choose a smoothing regularizer on both orientation (denoted ε^reg, o^) and e‐wave index (denoted ε^reg, n^) of the index ellipsoid. Note the regularization constraints encourage global smoothness during optimization while allowing to retrieve the local abrupt parameter changes in presence of singularities. The details on regularization and reconstruction are provided in Experimental Section and Methods and Notes S3 and S4 (Supporting Information).

### 2.3 Validation with Simulation

**Figure 2:**
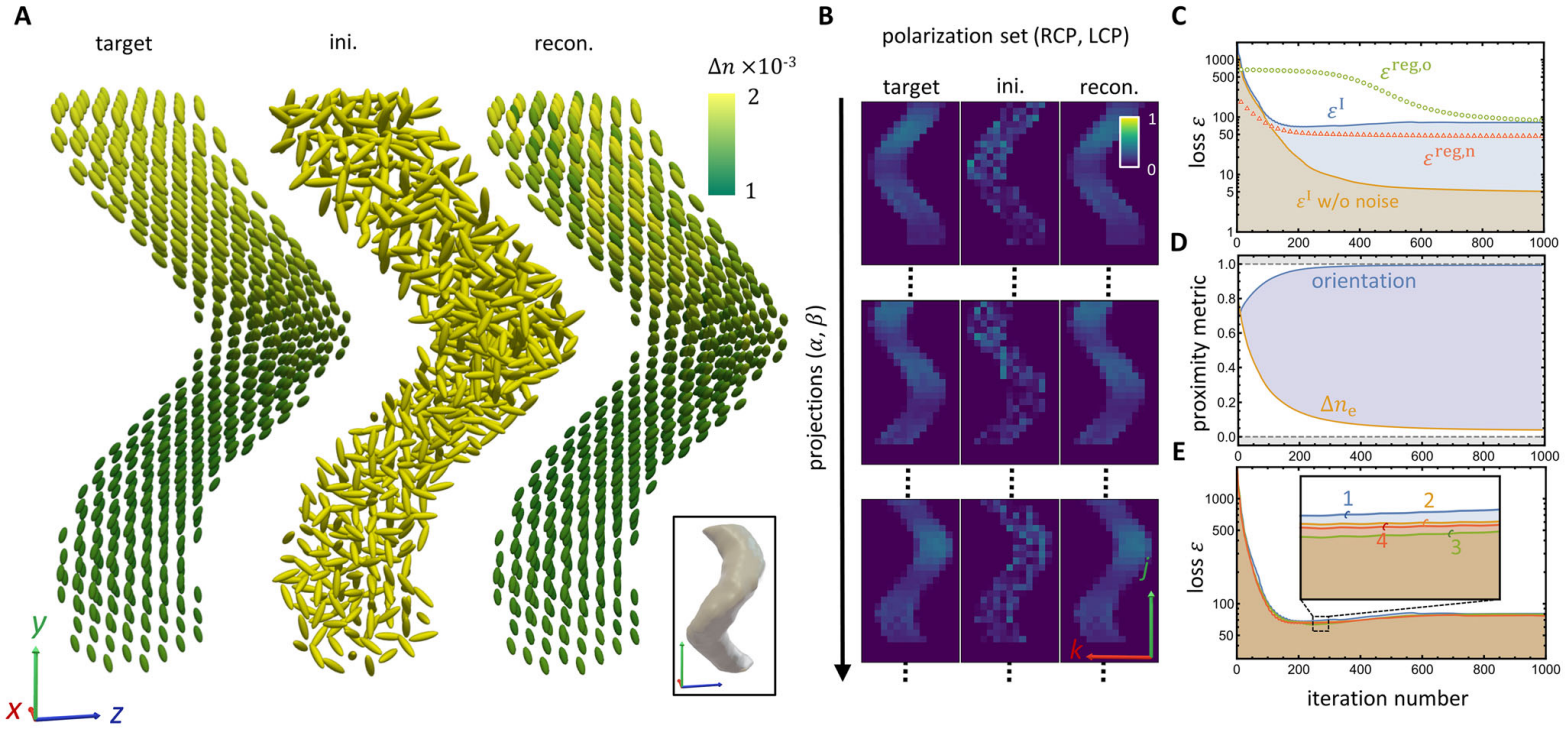
Numerical validation of TMPM. A birefringent right‐handed‐helix‐shape sample, as seen in the inset of (A), is utilized to verify TMPM. A) Index ellipsoid distributions for the target (ground truth), initial guess, and reconstructed results. The color gradient indicates the birefringence Δ*n*. B) Typical projections (1, 9, and 17) of the polarization set of PSG (RCP) and PSA (LCP) for the target, initial, and reconstructed results. Light intensity is normalized to its maximum. C) The optimization loss function for one fixed random initialization as a function of the reconstruction iteration. The error ε^I^, with Poisson noise (blue line), and the smoothing regularization terms for both the c‐axis orientation ε^reg, o^ (green circles) and e‐wave refractive index ε^reg,n^ (red triangles) are presented. As a comparison, the error ε^I^, recalculated without considering Poisson noise is displayed (orange line). D) The proximity of the solution to ground truth, specifically the orientation (calculated by dot product of unit vectors) and the extraordinary index *n*_e_ (calculated by absolute difference Δ*n*_e_), at each iteration. E) The convergence curves of the reconstruction with the initial parameter used in (C) and (D) (numbered as 1, with blue line), as well as another three different uniformly randomized initialization (numbered as 2–4, with green, red, and orange lines).

To validate the reconstruction strategy, a simulation model of a spiral‐shaped object of a size of 15 × 25 × 15 voxels, with a length of a single helical pitch of 22 voxels and a cross‐sectional radius of 3 voxels, as schematized in **Figure** **2**A (bottom right), is created. The orientations (c‐axis) in ground truth are in line with the helical trajectory. The birefringence Δ*n* has a gradual spatial variation range of [0.001, 0.002] for each voxel, and the absorption values within the range [0.1, 0.3], both of which are in the range of typical biological materials. The voxel size and the tomographic scan angles are maintained the same as in the experimental measurement to discuss. In addition, upon the projection images an experimentally realistic Poisson noise is applied to mimic the shot noise on a 12‐bit charge‐couple device (CCD) camera. Figure 2A showcases the target, the initial random guess, and the reconstructed result of the 3D index ellipsoid in each voxel, demonstrating the capability of the reconstruction method. The c‐axis orientation is almost perfectly rebuilt, with only slight distortion of Δ*n*, due to shot noise enforced. The projected images for the target, random initialization and reconstruction at different projections (1, 9, 17) are compared in Figure 2B for the polarization set of PSG (LCP) and PSA (RCP), showing the effectiveness of the method by the proximity between the reconstruction and the target. Some significant parameters are tracked through the optimization (Figure 2C), including the error metric ε^I^, the recalculated error using the referential projection with the shot noise removed, and the two regularization terms. The proximity to the target, i.e. ground truth, of the c‐axis orientation (blue line), calculated as the mean dot product of the target and the updated result through optimization, and their relative e‐wave index difference Δ*n*_e_ (orange line), normalised to the maximum birefringence Δ*n*  =  0.002, are illustrated in Figure 2D. The values of them respectively close to 1 and 0 showcase the reconstruction accuracy. To further test the optimization robustness, differently randomized initial parameters are applied, resulting in very similar convergences and reconstructions, as shown in Figure 2E.

### 2.4 Experimental Data Reconstruction of 3D Ultrastructure of Trabecular Bone

**Figure 3:**
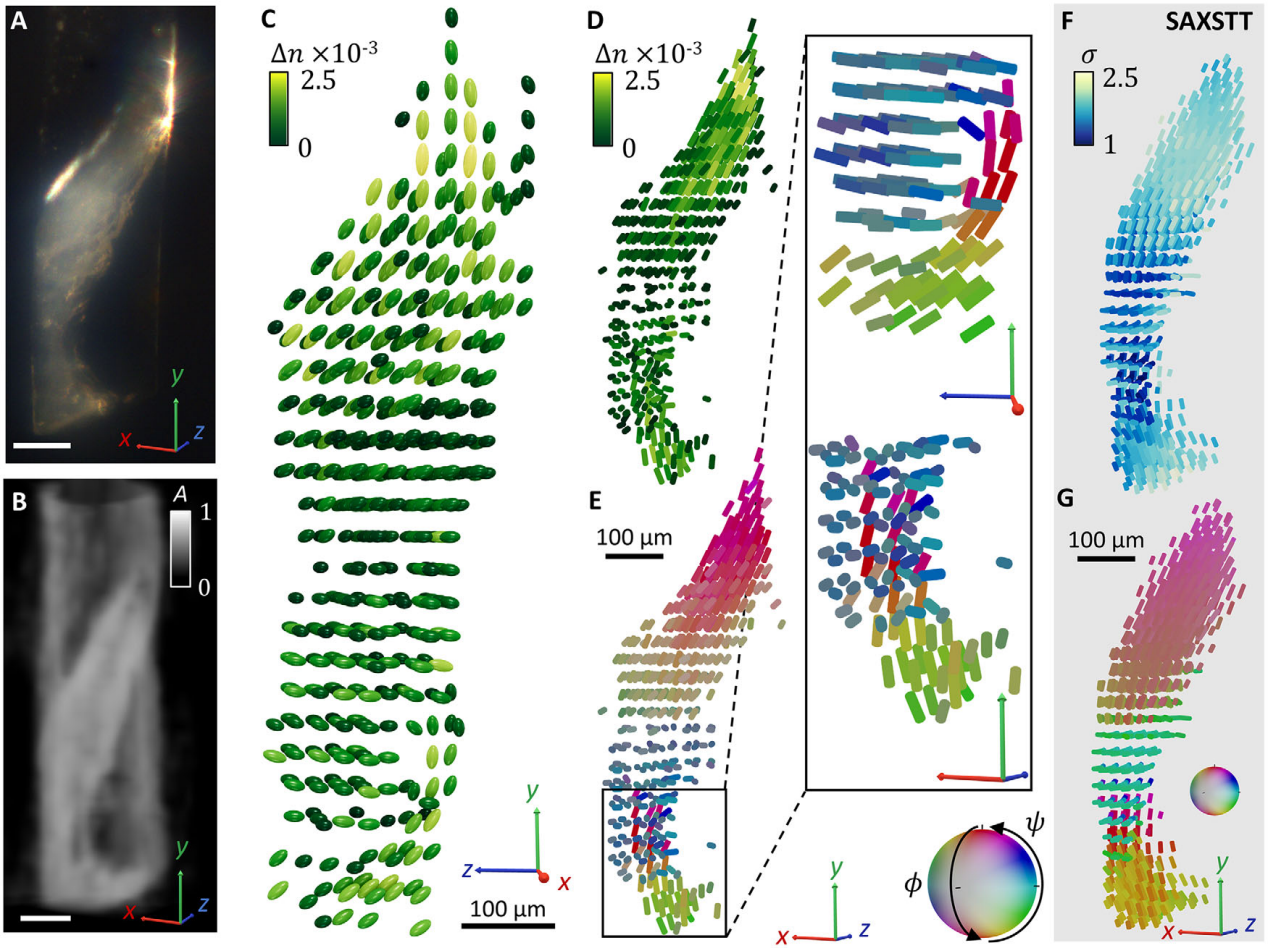
Reconstructed 3D optical anisotropy of trabecular bone. A) A microscope image of the bone sample immersed in oil under a lateral illumination (10 ×, 0.28 NA). B) Reconstructed tomogram of the absorption *A*. C) Reconstructed index ellipsoid in each voxel of the volume by TMPM. Both the ellipsoid length and the color gradient indicate the strength of birefringence Δ*n*, orienting its c‐axis. D and E) Other views of the reconstructed index ellipsoids, plotted as cylinders to highlight their c‐axis orientation, with a length proportional to the birefringence Δ*n*, color coded with (D) birefringence Δ*n* and (E) c‐axis angle (ϕ, ψ) where ψ is encoded on hue and ϕ in scale of the color value and saturation. Inset shows zoom‐in of the same region at two different views, outlining the c‐axis orientation. F and G) Nanostructure orientation reconstructed with SAXSTT, represented as cylinders following the structural orientation, with a length proportional to the degree of orientation and the scattered symmetric intensity, color‐coded with (F) the degree of orientation σ and (G) the 3D orientation angles (ϕ, ψ) following the same color reference as in (E).

To demonstrate the method, a piece of human trabecular bone was measured, embedded in polymethyl methacrylate (PMMA), and milled as a cylinder of roughly 1 mm length and 400 µm diameter (see Experimental Section and Methods for preparation details). An overview of the sample is presented in **Figure** **3**A as a microscopic image with lateral illumination. The bone displays 3D birefringence varying in amplitude and orientation, due to its collagen fiber densities and orientations. The measurement produced a total of ≈700 000 datapoints that notably significantly outnumbered the unknowns, ≈27 000, to ensure the solvability of the problem which was assisted by regularizers to avoid overfitting.

The absorption tomogram (Figure 3B) shows high absorbance at the interface of the PMMA and the surrounding oil, that is due to a residual lensing effect and surface roughness consecutive to the milling (without polishing) process. The 3D birefringence tomogram reconstructed with TMPM, that is the index ellipsoid in each voxel of the bone, is shown in Figure 3C with a color displaying the birefringence variations (see Movie S1, Supporting Information for an animated 3D view of the tomogram). Another view and representation of the full volume is shown in Figure 3D. Higher retardance values appear in the top and bottom of the sample, with the extracted ultrastructure orientation following the macroscopic orientation of the trabecular bone, while the middle part shows lower retardance values. Note the lower part of the sample consists of two overlaid domains of large difference in orientation (illustrated in the inset of Figure 3E), demonstrating the ability of TMPM to reconstruct 3D orientation variations within 3D macroscopic samples, which is impossible with 2D polarized light imaging approaches. This overlaid structure also shows TMPM's capability of detecting structural singularities in specimens, a key objective in microscopy for biomedical applications.

In order to validate the results of TMPM, the same sample was measured at similar spatial resolution (voxel size 25 µm) with SAXSTT^[^ ^21^ ^]^ as reference method. For bone, the measured birefringence is caused by the combination of intrinsic and form birefringence of the collagen fibers, SAXS on the other hand is sensitive to the electron density difference between the organic collagen and the mineralized hydroxyapatite. The SAXSTT reconstruction is shown in Figure 3F, with degree of orientation color‐coded, and Figure 3G with color‐coding of the orientation angles (ϕ, ψ). The SAXSTT reconstructed volume does not exhibit exactly the same shape as TMPM, likely caused by the optical retardance and the X‐ray scattering measurement not having the same sensitivity as well as some remaining lensing effects at the sample surfaces in the context of optical light. However, the 3D orientation of mineralized collagen fibre obtained from SAXSTT match the reconstructed ultrastructure orientation from TMPM rather well, except from the middle part, where low retardance values (Figure 3C,D) as well as low degree of orientation values (Figure 3F) indicate that there is a less uniform orientation of collagen fibers within the voxels.

**Figure 4:**
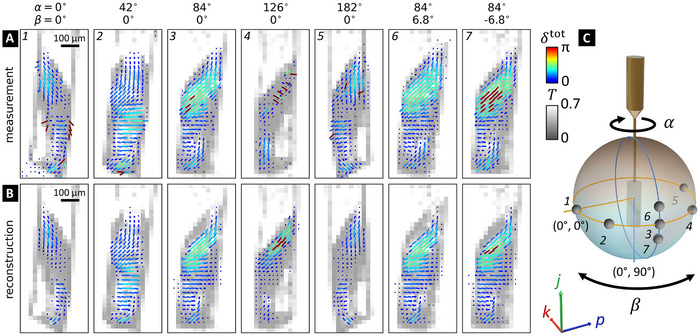
Projected optical retardance and fast‐axis angle of trabecular bone. The fast‐axis angle θ^tot^ and total linear retardance δ^tot^ tick‐maps from A) the measured projections compared to B) the simulated projections from the TMPM reconstruction. The isotropic transmission (grey background regions) extracted from the data is displayed to highlight the sample edges. 7 projections at different rotation angles α and tilt angles β as illustrated in (C), represented as grey points on the unit sphere, are shown. The tick direction in the maps indicates the fast‐axis angle θ^tot^ while both the cylinder length and the color stand for the optical retardance δ^tot^.

Measured projections, shown in **Figure** **4**A, are compared to simulated projections from the TMPM reconstruction (Figure 4B) at 7 different tomographic angles α and β (Figure 4C), using the total linear retardance δ^tot^ and fast‐axis orientation θ^tot^ integrating through the sample thickness, extracted from their Müller matrices.^[^ ^32^ ^]^ Overall, there is a good match between the retardance and fast‐axis angle between the reconstruction and the measurement. Some small mismatches in shape, δ^tot^, and θ^tot^ are most likely caused by the high attenuation and lensing effect of the sample, incurring inaccurate extraction from measurement data rather than TMPM reconstruction. One can also notice that the measurements display some unexpected high birefringence at some interfaces (Projection 1 in Figure 4A for example), most probably also originating from unwanted Fresnel scatterings at the sample edge that our model try to erase. These differences at edges also explain the remaining disagreement of the sample shapes between TMPM (Figure 3D,E), with SAXSTT reconstruction (Figure 3F,G).

## 3 Conclusion

We demonstrated TMPM, a novel 3D structural imaging approach based on polarimetric intensity measurements. The new method treats polarization‐propagation problem of high non‐linearity as a regularized least square one to retrieve the index ellipsoid of the sample at each voxel. TMPM reveals the 3D ultrastructure of linearly birefringent biological materials at microscopic resolution, without incorporating complicated phase measurement, offering advantages of high accessibility and low cost of the experimental setup. The complete physical model and optimization algorithm are verified using a simulation example and experimental measurement of a human trabecular bone piece, obtaining a convincing agreement. The hierarchical microstructure of the 3D trabecular bone sample was successfully retrieved, reflecting its collagen fiber distribution and orientation,^[^ ^33^ ^]^ agreeing with conventional 2D polarimetric image analysis measured at several projections as well as with the reference using SAXSTT.

The pathway to improve the technique is threefold, encompassing sample pre‐processing, experimental setup improvement and physical‐model refinement. First, as for visible light microscopy, the sample must be transparent, presenting negligible scattering. To arrange this for dense and complex biological samples, tissue clearing process can be used. As well, since TMPM assumes a ballistic light propagation, the sample needs to have a slow‐varying refractive index to avoid significant ray reflection (typically < 10^−2^) and small birefringence to minimize double refraction (typically < 10^−2^). Fortunately, this assumption applies to a broad range of material from model organisms such as Zebrafish,^[^ ^34^, ^35^, ^36^ ^]^ and C. Elegans,^[^ ^37^ ^]^ biomineralized materials,^[^ ^38^ ^]^ human connective tissues,^[^ ^39^ ^]^ to even larger specimens such as full mouse brain^[^ ^40^ ^]^ and mouse embryo.^[^ ^41^ ^]^ On that note, the bone sample presented in this work was specially chosen to be able to validate the obtained 3D orientation with the more established SAXSTT, creating an extra challenge for the technique, as the sample presented limited transparency. It proves however, that the method can be conveniently extended to diverse biological samples. Second, the experimental setup is very sensitive in measurement to sample‐goniometer misalignment and mechanical vibrations, as the sample needs to be always in focus. The current solution was to sacrifice the resolution of measurements to increase the depth of field. However, high resolution remains a practical need, and one may use a robotic arm instead of a manual goniometer to perform the tomographic acquisition, allowing a fine control over the sample position in the focal plane. Alternatively, the use of metalens‐based objectives^[^ ^42^, ^43^ ^]^ can be considered to increase the depth of field for resolution improvement, whilst precise index‐matching of environmental liquid should be introduced to maximize the cancellation of lensing effects.^[^ ^44^, ^45^ ^]^ At last, the TMPM model could be extended to incorporate other interesting polarimetric properties such as diattenuation/dichroism, biaxial anisotropy and depolarization, covering in this way more complex samples and more subtle structural characterizations.

To conclude, this novel polarized‐light approach, leveraging numerical optimization to reconstruct sample's 3D structural information, offers a convenient experimental method for optical characterization and detection that often requires complicated phase measurement, and creates a more accurate understanding of sample materials of non‐homogenous structures that cannot be approximated with a thin anisotropic layer. It opens up an exciting opportunity for the non‐destructive study of hierarchical materials, to understand the functional properties conveyed by the 3D alignment of ultrastructure. It combines the advantages of tomographic methods being able to study 3D structure in a non‐destructive way, with the advantages of optical methods in respect of easy accessibility as well as not inducing radiation damage, a common problem in related X‐ray methods. This also opens its use for in‐vivo studies of model organisms,^[^ ^34^, ^35^, ^36^, ^37^ ^]^ following development stages or progress of diseases,^[^ ^12^, ^13^, ^14^, ^15^, ^40^, ^41^ ^]^ or allows for undisturbed in situ mechanical tests.

## 4 Experimental Section

### Ballistic Regime

The model assumes ballistic light propagation, where photons travel along straight paths through the sample with negligible scattering. A quasi‐ballistic regime is commonly employed as an approximation, applicable when the sample thickness falls between the scattering mean free path (typically ≈0.1 mm) and the transport mean free path (typically ≈1 mm).^[^ ^46^ ^]^ While these values show significant sample‐dependent variation and the approximation becomes less accurate under strong scattering conditions, they confirm that the ballistic assumption holds for biological samples up to several hundred microns thick. Furthermore, TMPM ensures non‐ballistic photons are discarded in the measurements by using a spatial frequency low‐pass filter, i.e. a low numerical aperture objective, and in the reconstruction by using a tomographic approach based on the Radon transform.^[^ ^46^ ^]^

### Linear Birefringent Voxel and Forward Model

The key strategy of TMPM is to model the optical properties in each sub‐volume or voxel of the tomogram as a linear birefringent crystal. Light ray traveling through a linear birefringent crystal typically divides into two, that is the o‐ and e‐waves, which is known as double refraction, forming two wave normal surfaces.^[^ ^22^ ^]^ The spherical surface of the o‐wave leads to a constant effective refractive index *n*_o_, independent on incidence light angle. By contrast, the e‐wave has an ellipsoidal normal surface, with an index *n*_E_ subject to light ray direction (forming an index ellipsoid), expressed as^[^ ^22^ ^]^

(1) $$n_{E}\left(n_{o},\;n_{e},\;c,\;\alpha ,\;\beta \right)=\frac{n_{o}n_{e}}{\sqrt{n_{o}^{2}\mathrm{si}n^{2}\varphi +n_{e}^{2}\mathrm{co}s^{2}\varphi }}\;$$

where φ is the angle between the c‐axis and the direction of the light. This angle can be calculated in the following way:

(2) $$\varphi \left(c,\;\alpha ,\;\beta \right)=\arccos\left(p\cdot R_{s}c\right)\;$$

where **p** is light propagation unit vector along *p* axis, **c**  = (sin ϕcos ψ,  sin ϕsin ψ,  cos ϕ)  the c‐axis orientation unit vector in the object referential frame (*x*, *y*, *z*), · the scalar product and *R_s_* the rotation operator between the lab referential (*k*, *j*, *p*) and the object referential (*x*, *y*, *z*)

(3) $$R_{s}=R_{k}\;\left(\beta \right)R_{j}\left(\alpha \right)$$

with *R_k_* and *R_j_* the 3D rotation matrices around the axes *k* and *j*. Similarly, the angle θ between the projected c‐axis on the transverse plane of the light and the *k* axis, known as fast axis orientation, is calculated as follow:

(4) $$\theta \left(c,\;\alpha ,\;\beta \right)=\arccos\left(k\cdot \hat{P}\left(R_{s}c\right)\right)\;$$

where **k**  = (1, 0, 0)* *, and $\hat{P}$ is the projection operator onto the transverse plane of the light direction (i.e. transverse to **p**). The different incidence produces different optical paths and e‐wave index *n*_E_, giving rise to the different phase advances for the orthogonal polarization states of o‐ and e‐waves, with the phase difference known as retardance δ(*n*_o_, *n*_e_, **c**,  α,  β)  =  2π*d*Δ*n*_E_/λ, where Δ*n*_E_(*n*_o_, *n*_e_, **c**,  α,  β)  = *n*_E_ (*n*_o_, *n*_e_, **c**,  α,  β) − *n*_o_ is the birefringence produced, *d* the ray path length through the voxel, and λ the light wavelength. More specifically, since the birefringence Δ*n*  = *n*_e_  − *n*_o_ dominates the polarimetric behavior rather than the two absolute refractive indices when Δ*n* is small (see Note S2, Supporting Information, and the small birefringence assumption applies to most biological materials), *n*_o_ is assumed to be a known identical to that of index‐matching liquid used without losing accuracy for retrieving other parameters (ϕ, ψ,  *n*_e_). For clarity of writing, the parameters δ, φ,  and θ will be expressed without their dependent parameters in the following text and equations. The Müller matrix of a linearly birefringent crystal, described as a linear retarder, takes the form of^[^ ^8^ ^]^

(5) $$M_{\mathrm{LR}}=t_{\mathrm{LR}}\;M_{R}\left(-\theta \right)M_{\mathrm{LR}}^{0}\left(\delta \right)M_{R}\left(\theta \right)$$

where *t*_LR_ stand for a scalar value of the transmission coefficient, and *M*_R_(θ) and $M_{\mathrm{LR}}^{0}\left(\delta \right)$ are the Müller matrices of an optical rotator and an unrotated linear retarder, respectively written as

(6) $$M_{R}\;\left(\theta \right)=\left[\begin{array}{cccc} 1 & 0 & 0 & 0\\ 0 & \cos2\theta & \sin2\theta & 0\\ 0 & -\sin2\theta & \cos2\theta & 0\\ 0 & 0 & 0 & 1 \end{array}\right]\;,M_{\mathrm{LR}}^{0}\;\left(\delta \right)=\left[\begin{array}{cccc} 1 & 0 & 0 & 0\\ 0 & 1 & 0 & 0\\ 0 & 0 & \cos\delta & \sin\delta \\ 0 & 0 & -\sin\delta & \cos\delta \end{array}\right]\;$$

This leads to a Müller matrix at each voxel with a parameter space for the reconstruction (ϕ, ψ,  *n*_e_) expressed as:

(7) $$\begin{array}{ccc} & & M_{\left(n_{e},\phi ,\psi ,\alpha ,\beta \right)}^{p}\\ & & \;=t_{\mathrm{LR}}\left[\begin{array}{cccc} 1 & 0 & 0 & 0\\ 0 & \mathrm{co}s^{2}2\theta +\cos\delta \mathrm{si}n^{2}2\theta & \left(1-\cos\delta \right)\cos2\theta \sin2\theta & -\sin\delta \sin2\theta \\ 0 & \left(1-\cos\delta \right)\cos2\theta \sin2\theta & \cos\delta \mathrm{co}s^{2}2\theta +\mathrm{si}n^{2}2\theta & \cos2\theta \sin\delta \\ 0 & \sin\delta \sin2\theta & -\cos2\theta \sin\delta & \cos\delta \end{array}\right] \end{array}$$

Note that *n_e_*, ϕ and ψ depend on the *p* ^th^ voxel alongside the *p* axis but are indicated without expressing this dependency for the sake of readability. Since the measured Müller matrix *M*_(α,β)_ of the projection (α, β) at pixel (*k*, *j*) can be considered as the product of the Müller matrices of all voxels in the light path, obtain the following Equation (8)

(8) $$M_{\left(\alpha ,\beta \right)}\;\left(k,j\right)=\prod_{p}^{N\left(k,j\right)\;}M_{\left(n_{e},\;\phi ,\psi ,\;\alpha ,\;\beta \right)}^{p}\left(k,j\right)$$

where *N*(*k*, *j*) is the number of ordered voxels in the path between the source and the (*k*, *j*) pixel on the sensor. The simulated intensity at the sensor can be calculated using:

(9) $${\hat{I}}_{li}\;\left(k,j\right)=S^{T}\;M_{l}^{\mathrm{PSA}}M_{\left(\alpha ,\beta \right)}\left(k,j\right)M_{l}^{\mathrm{PSG}}S$$

where the column Stokes vector of unpolarized light **S**  =  (1, 0, 0, 0) and T symbolizes the transpose. $M_{l}^{\mathrm{PSG}}$ and $M_{l}^{\mathrm{PSA}}$ are the Müller matrices of PSG and PSA of the *l* ^th^ polarization set out of the 16, and *i* the projection index corresponding to the tomographic angles (α, β).

### Optimization Parameters and Gradients

A gradient descent optimization algorithm is used to minimize the error between the measured intensities and the simulated intensities with the metric:

(10) $$\varepsilon^{I}=\sum_{i}\sum_{l}\sum_{j,k}{\left({\hat{I}}_{li}\left(k,j\right)-I_{li}\left(k,j\right)\right)}^{2}\;$$

where *I_li_* (*k*,*j*) and ${\hat{I}}_{li}\left(k,j\right)$ stand for the measured and simulated intensities, respectively, along a ray path at the pixel (*k*,  *j*) in the image of the *i* ^th^ projection with the *l* ^th^ polarization set. See Note S5 and Figure S3 (Supporting Information) for an example of the measured and reconstructed intensities of a selected projection.

To minimize the error ε^I^, we develop its gradient function with respect to the three searched parameters {ϕ, ψ,  *n*_e_} of a certain voxel, with $u\in \{\phi ,\psi ,\;n_{e}\}$ as a universal denotation:

(11) $$\begin{array}{ccc} \frac{\partial \varepsilon^{I}}{\partial u} & = & \sum_{i,l,k,j,p}2\left({\hat{I}}_{li}\left(k,j\right)-I_{li}\left(k,j\right)\right)\sum_{a,b=1}^{4}\\ & & \times \;{\left[\left(Q⊗V\right)⊙\left(\frac{\partial M_{ikj,p}^{\mathrm{sam}}}{\partial \theta }\frac{\partial \theta }{\partial u}+\frac{\partial M_{ikj,p}^{\mathrm{sam}}}{\partial \delta }\frac{\partial \delta }{\partial u}\right)\right]}_{ab} \end{array}$$

where **Q** and **V** represent the polarization state vectors after and before the *p* ^th^ voxel in the path landing at the sensor pixel (*k*, *j*) in the *i* ^th^ projection, $Q=S^{T}\;M_{l}^{\mathrm{PSA}}O_{ikj,p}$ and $V=L_{ikj,p}\;M_{l}^{\mathrm{PSG}}S$ where *O*_*ikj*,*p*_ and *L*_*ikj*,*p*_ represent the Müller matrix products of the voxels after and before the *p* ^th^ one $M_{ikj,p}^{\mathrm{sam}}$. ⊗ and ⊙ stand for the tensor and Hadamard products. Note (Equation 11) incorporates a large quantity of sum terms, most of which vanish when $M_{ikj,p}^{\mathrm{sam}}$ does not depend on the parameter $u\in \{\phi ,\psi ,\;n_{e}\}$.

To speed up the reconstruction, we employed the Nesterov accelerated algorithms,^[^ ^20^ ^]^ using momentum‐based stochastic descent gradient, to quickly minimize the Lipschitz‐continuous loss function by leaping over the large number of local minima. To further improve the convergence speed and precision, a stage‐wise optimization is combined in use, by alternating the optimization of the c‐axis orientation angles (ϕ, ψ) and the e‐wave index *n*_e_ (see Note S4, Supporting Information). Smoothing regularizers, specifically for the enforcement of spatially slow changes of separately e‐wave index and c‐axis vector, is applied as penalty terms (respectively ε^reg,n^ and ε^reg,o^) to impose a proper level of global smoothness of the optimised parameters (ϕ, ψ,  *n*_e_), and are demonstrated to be very useful in convergence of loss function ε^I^ and reconstruction accuracy. The reconstructions were carried out in a computer of central processing unit (CPU, Intel(R) Core(TM) i7‐10700), with CPU‐based shared‐memory multiprocessing parallelism implemented. The time consumes typically ≈35 min for 100 iterations for the bone reconstruction, with apparent potential for optimization‐speed boost with better computing power.

### Bone Sample Preparation

A trabecula from a T12 human vertebra from a 73‐year‐old man was extracted and cleaned from soft tissue.^[^ ^21^ ^]^ The human vertebra had been obtained from the Department of Anatomy, Histology, and Embryology at the Innsbruck Medical University, Innsbruck, Austria, with the written consent of the donor according to Austrian law. All subsequent procedures were in accordance with Swiss law, the Guideline on Bio‐Banking of the Swiss Academy of the Medical Sciences (2006) and the Swiss ordinance 814.912 (2012) on the contained use of organisms.

The trabecular bone sample was embedded in poly‐methyl methacrylate (PMMA) that allows for high transparency and optical isotropy to avoid additional optical distortions, and then milled to a length of ≈0.8 mm and diameter ≈0.2 mm. The milled sample was mounted on the head of a thin metal needle using epoxy resin glue, measured within TMPM and small‐angle X‐ray scattering tensor tomography.

### Experimental Setup and Measurement

Light from an incoherent narrow bandwidth red light source (Thorlabs MCS103, 625 nm) was used, modulated by PSG, consisting of a linear polarizer LP (Newport 10LP‐VIS‐B), a half‐wave plate HWP (Newport 10RP32‐632.8) and a quarter‐wave plate QWP (Newport 10RP04‐24), and analyzed by PSA that has a symmetric set of QWP and LP. QWPs and LPs are all mounted on rotating stages (Thorlabs K10CR1/M) to enable a flexible polarization modulation. The sample, immersed into an index‐matching oil of refractive index 1.48 (BioChemica) to minimize the edge refraction and scattering and increase the depth of field, is mounted on a homemade goniometer stage (Edmund Optics 55–839 and 55–028) to perform the angular tomographic scan. The transmitted polarized light is collected by a ×2 objective lens (Mitutoyo 378‐801‐12, NA 0.055), a tube lens (Thorlabs TTL200‐A) and a CCD camera (IDS U3‐3060CP‐M‐GL, 1920 × 1200 pixels, 5.86‐µm pixel size, 12 bits). See Note S1 (Supporting Information) for more details on the setup.

For the measurement of the bone sample, a tomographic scan was performed with Müller polarimetric measurement, on the previously described setup. For the tomographic scan, rotation angles of $\Delta \alpha =14^{\circ }$ from 0° to 360° for tilt angles of $\beta =0^{\circ }$ and $\pm 6.8^{\circ }$ were measured, generating 78 projections and 1248 polarimetric projection images in total that are acquired by the CCD camera at a resolution of 2.93 µm. In order to reach the required axial field depth of the bone sample at all projection angles, the images were downsampled by 8 times, lowering our resolution to 23.44 µm and a full field of view of 240*150 pixels (≈820 × 375 µm^2^) (see Note S6, Supporting Information for details on the depth of field). The milled trabecular bone sample and surrounding PMMA occupy a volumetric space of ≈375 × 820 × 375 µm^3^ (shown as microscopic image in Figure 3A and the absorption tomogram in Figure 3B). Considering the voxel size same as the measurement resolution, the acquisition ends up with 698 880 measured datapoints for 26 880 parameters to optimize (35 840 including the absorption).

To correct for the misalignment of the projection images, a software alignment with subpixel accuracy was employed onto the projections using the absorption information to quantify the sample shifts in the images as compensations to the positioning errors^[^ ^47^ ^]^ (see Note S6, Supporting Information).

### SAXSTT Measurement and Reconstruction

The sample was measured by SASTT at the cSAXS beamline of the Swiss Light Source (SLS) at the Paul Scherrer Institut (PSI), Switzerland. The X‐ray beam energy was set to 12.4 keV and the beam had a full width at half maximum of 24 × 16 µm^2^. The sample was raster scanned using a step size of 25 µm in both the horizontal and vertical directions, resulting in a total of 77 projections measured at various tilt (0,  15,  30°) and rotation angles (0 − 180° for the 0° tilt, 0 − 360° for the others, steps of 16,  36°). The scattered photons were measured by a Pilatus 2M detector placed 2.13m downstream of the sample. A 2‐meter flight tube was placed between the sample and the detector to reduce air scattering. The direct beam was blocked by a 1.5‐millimeter steel beamstop inside the flight tube. The intensity of the transmitted beam was measured from the fluorescence signal from the beamstop, using a Cyberstar (Oxford Danfysik) detector.

The mineralized collagen fiber orientation was reconstructed from the background subtracted collagen peak (scattering vector *q* within the range (0.09, 0.115) nm^−1^) using the software package *Mumott* (version 1.2).^[^ ^48^ ^]^ The projections acquired at non‐zero tilt angles were aligned to the zero‐tilt tomogram by using sub‐pixel registration algorithms.^[^ ^47^ ^]^ The reciprocal space maps were reconstructed in each voxel using a basis set of even‐ordered spherical harmonics (orders 0,2,4, and 6). The optimization problem was formulated using a quadratic loss function with a Laplacian regularizer and solved using a L‐BFGS algorithm.^[^ ^49^ ^]^ In the final step, the fiber orientation was extracted by eigenvector analysis of the 2^nd^ order spherical harmonics, yielding a main orientation and degree of anisotropy.

## Conflict of Interest

The authors declare no conflict of interest.

## Author Contributions

Y.C. and A.B. contributed equally to this work. Y.C. and A.B. designed the TMPM approach, conducted the TMPM and SAXSTT experimental measurement, and developed the reconstruction algorithms, Arthur Baroni designed and constructed the experimental setup, Torne Tänzer conducted the SAXSTT analysis, Leonard Nielsen helped with the TMPM algorithm development and TMPM experimental measurement. Marianne Liebi conceived and supervised this research project, conducted the SAXSTT measurement. Y.C., A.B., and M.L. wrote the paper and all co‐authors have contributed toward the final draft.

## Supporting information



Supporting Information

Supplemental Movie 1

## Data Availability

The data that support the findings of this study are openly available in Zenodo at https://doi.org/10.5281/zenodo.14043816, reference number 14043816. The reconstruction codes are openly available in Zenodo at https://doi.org/10.5281/zenodo.15309835, reference number 15309835.
