# Supplementary material for: Reconstructing Three‐Dimensional Optical Anisotropy with Tomographic Müller‐Polarimetric Microscopy
Source: Adv Sci (Weinh). 2025 May 8;12(27):2502075. doi: 10.1002/advs.202502075 (PMC12279228; doi:10.1002/advs.202502075)
Supplement: Supplementary file 1 — Supporting Information [file ADVS-12-2502075-s002.docx]

Supporting Information

Reconstructing three-dimensional optical anisotropy with tomographic Müller-polarimetric microscopy

Yang Chen,* Arthur Baroni,* Torne Tänzer, Leonard Nielsen, Marianne Liebi*

**Table of Contents**

1. Supporting Information text (Note S1 to S6)
2. Supporting Information figures (Figure S1 to S5)
3. Legend for Supporting Information movies (Movie S1)

1. Supporting Information text

**Note S1. Experimental setup**

The experimental system is a combination of a free-space polarimeter with a goniometer to hold the sample, introduced schematically in Figure 1, and detailed in Figure S1. It uses an incoherent narrow bandwidth red light source (non-polarized diode at 625nm, Thorlabs MCS103), coupled with an optical fiber (Thorlabs M122L02) and collimated with a collimator (Thorlabs PAF2P-A15A). The polarization of the probe in PSG is controlled by a linear polarizer, PSG LP (Newport - 10LP-VIS-B), at $0^{^{\circ}}$ to enforce a linear state of the source, followed by a half-wave plate, PSG HWP (Newport 10RP32-632.8), on a rotating stage (Thorlabs K10CR1/M, $\theta_{1}$) to control its orientation, and a quarter-wave plate, PSG QWP (Newport 10RP04-24), on another rotating stage ($\theta_{2}$) to control its ellipticity.

The sample is mounted on a custom-build goniometer stage, composed of a one-axis goniometer (Edmund Optics 55-839) for tilts and a rotary stage (Edmund Optics 55-028) for rotations, to perform the angular tomographic scans. In addition, the sample is immersed into a cuvette (Portmann UG-752, 30mm OPL, clear border and optical glass) with index matching liquid (immersion oil, BioChemica – A3494,0500) at n=1.48 to minimize scattering and increase the depth of field.

The exit light is collected by an objective (which can be changed depending on the resolution required). In the presented example it is one of magnification M = 2 and numerical aperture NA = 0.055, and Mitutoyo Plan Apo Infinity Corrected (Mitutoyo 378-801-12).

The polarization analysis is performed by PSA between the objective (Mitutoyo 378-801-12, NA 0.055) and its associated tube lens (Thorlabs - TTL200-A) to have parallel beams and to avoid angular artefacts on the polarization elements. The polarization states to analyze the sample are arranged in a symmetrical way to those of PSG with a quarter wave plate, PSA QWP, on a rotating stage ($\theta_{3}$) followed by a linear polarizer, PSA LP, on another rotating stage ($\theta_{4}$). The sensor, a CCD camera of 12bits 1920x1200 pixels with 5.86-µm square pitch (IDS U3-3060CP-M-GL), is placed at the image plane of the tube lens to collect the resulting intensity images.

The data were acquired on the setup in Figure S1, using an exposure of 50 ms (to use the maximum of the camera dynamic without saturating it) for each projection. Due to the objective magnification and the sensor pixel pitch, the acquisition was performed at a resolution of $2.93 \text{μ}\text{m}$, field of view of $\text{FoV}=5625.6\times3516 {\text{μ}\text{m}}^{\text{2}}$ and a depth of field of $\text{DoF}= 310 \text{μ}\text{m}$. For each projection we performed a polarimetric scan using sixteen combinations of PSG [LP $0^{^{\circ}}$, LP ${45}^{^{\circ}}$, RCP, LCP] and PSA [LP $0^{^{\circ}}$, LP ${45}^{^{\circ}}$, RCP, LCP]. For the tomographic scan we choose to use a rotation step of ${14}^{^{\circ}}$ ranging from $0^{^{\circ}}$ to ${354}^{^{\circ}}$ and a tilt step of ${6.8}^{^{\circ}}$ ranging from ${-6.8}^{^{\circ}}$ to ${+6.8}^{^{\circ}}$. This results in 3 tilts and 26 rotations per tilt, in summary 78 projections for a total of 1248 intensity images.

**Note S2: Refractive indices and effective birefringence approximation**

In Equation 1 in Experimental Section and Methods, we have seen that for a birefringent material the effective refractive index $n_{\text{E}}$ perceived by the light is dependent on the ordinary index $n_{\text{o}}$, extraordinary index $n_{\text{e}}$ and the angle $\varphi$ between the material c-axis and direction of the light. Since the retardance that we want to retrieve at every reconstructed voxel is defined for birefringent positive uniaxial material as $\Delta n= n_{\text{e}}- n_{\text{o}}$, one can see that we would need to reconstruct or to approximate very precisely either $n_{\text{e}}$ or $n_{\text{o}}$. Nevertheless, for most biological samples the retardance is very low, i.e. $\Delta n\ll1$. This leads to an elegant simplification of the effective (i.e. perceived) birefringence $\Delta n_{\text{E}}$, explicitly from^[1,2]^

|  | $\Delta n_{\text{E}}=\frac{n_{o}n_{e}}{\sqrt{n_{o}^{2}\sin^{2}\varphi+n_{e}^{2}\cos^{2}\varphi}}- n_{\text{o}},$ | (S1) |
| --- | --- | --- |

to

|  | $\Delta n_{\text{E}}= \Delta n \sin^{2}\varphi$. | (S2) |
| --- | --- | --- |

This approximation^[3]^, very precise for low retardance, prove that the retardance $\Delta n$ can be retrieved from the effective birefringence $\Delta n_{\text{E}}$ at different angle even without knowing the refractive indices. This allows us to approximate the ordinary refractive index $n_{\text{o}}$ in our analysis without problematic loss of precision on the reconstructed birefringence.

**Note S3: Regularization**

The sample is considered to have collective structural features (birefringence and optical-axis orientation), allowing quicker convergence with descriptive regularizers. In terms of the orientation, we use the cross product between the unit orientation vectors of neighboring voxels to quantify the structural correlation between them. It is standard practice that the correlation is weighted using the volumetric distance between the two voxels, with the regularization term $\varepsilon^{reg,o}$cast as

|  | $\varepsilon^{reg,o}=\sum_{i,j} {w_{ij}}^{2}\left( \hat{n}_{i}\times\hat{n}_{j} \right)^{2}, w_{ij}=\frac{1}{\left\vert\mathbf{r}_{i}-\mathbf{r}_{j} \right\vert},$ | (S3) |
| --- | --- | --- |

where $\hat{n}_{i/j}$ is the unit orientation vector of the $i$^th^ and $j$^th^ voxels, $\mathbf{r}_{i/j}$ the volume position vectors of the two voxels and $w_{ij}$ the weight.

In the case of birefringence, the adiabatic variation of the e-wave refractive index is also incorporated, with similar weighting but on the index difference between voxels, leading to the use of regularization term $\varepsilon^{reg,n}$ as

|  | $\varepsilon^{reg,n}=\sum_{i,j} {w_{ij}}^{2}\left( n_{e,i}-n_{e,j} \right)^{2},$ | (S4) |
| --- | --- | --- |

where $n_{e,i/j}$ is the e-wave index of the voxel $i/j$.

**Note S4: Reconstruction framework**

The measurement data is fed into an alignment algorithm (see Note S6) to register the projections at different tomographic angles. With the known projection parameters, the ray tracing is performed, ending up with fixed optical paths through optimization (see also in the main text). After that, the result, together with guessed optimization parameters ($\phi,\psi, n_{e}$), is utilized to calculate the Müller matrix, and then the error metric $\varepsilon^{I}$ (see Experimental Section and Methods in the main text), while ($\phi,\psi, n_{e}$) is used also to compute the smoothing regularisers $\varepsilon^{reg,o}$ and $\varepsilon^{reg,n}$. In the end, the loss function $\varepsilon$ is calculated using

|  | $\varepsilon=\varepsilon^{I}+\lambda_{o}\varepsilon^{reg,o}+\lambda_{n}\varepsilon^{reg,n},$ | (S5) |
| --- | --- | --- |

where $\lambda_{o/n}$ denotes the regularization coefficients for $\varepsilon^{reg,o/n}$.

We applied stage-wise optimization, i.e. alternating the implementation of the two regularizers, by setting $\lambda_{o}$ or $\lambda_{n}$ to be 0 for only enforcing the parameters under optimization. The framework of the reconstruction is showcased in Figure S2.

**Note S5: Tomographic projections**

Here we present the full set of measured intensities $I$ for the projection number 7, at $\left( \alpha,\beta\right)=({84}^{^{\circ}},0^{^{\circ}})$, to show full extent of the raw data, and the reconstructed projections, in Figure S3**A**. Note that the TMPM optimization expects to deal with the variation of intensity originating from the sample’s polarization effects, rather than the isotropic absorption. To achieve that, the isotropic transmittance *t* is extracted from the 2D intensity data as exampled in Figure S3**B**, as a multiplicative cumulation of $t_{\mathrm{LR}}$ for all the birefringent voxels in the ray path, and used to scale the corresponding ray-path intensity simulated (see $\hat{I}_{li}\left( k,j \right)$ in Equation 9 of the main text). The intensity maps by removing the transmittance are displayed in Figure S3**C**.

**Note S6: Alignment of the projections**

With the measurement strategy, i.e., tomography, the sample should be considered as rotating over its own long axis (*y*-axis) passing through its center. Since the goniometer used was operated by hand any vibration and difference of alignment between the sample’s axis and the goniometer’s axis creates a significant translational shift of the sample, and it was unable to compensate this misalignment mechanically. Therefore, we carried out an alignment algorithm to correct this shift numerically, based on the work of Odstrčil *et al.* ^[4]^. These shifts are easily visible on Figure S4**A** with a magnitude around $500 \mu m$. The shift along *x*-axis follows a sinusoidal behavior typical for an off-axis sample, with some abrupt change linked to change of tilt and shock during manipulation. The *y*-axis one almost plateaus for each tilt, which is common for a sample lying a bit away from the goniometer rotation center. The field of view for the sinogram is displayed in Figure S4**B** for the first projection, with the sample observed at the point of its tip that hold it. One can easily perceive its slight tilt, and the tip itself is off-centered in reference to the goniometer. The sinograms before and after the numerical alignment calculation are compared in Figure S4**C**, through the 2D cut indicated with the dashed line in Figure S3**B**, where one can clearly see the unwanted shifts on the unaligned sinogram, and how they are removed in the alignment optimization.

We schematically introduce in Figure S5 the link between the depth of field $\text{DoF}$ and the tomographic experiment, in the case of a sample aligned (Figure S5**A**) and unaligned with the goniometer (Figure S5**B**). On the aligned case (Figure S5**A**), the $\text{DoF}_{\text{geometry}}$ needed is dependent on the sample geometry (diameter $d$ and length $l$), the refractive index of the medium $n_{\text{oil}}$ and the maximum tilt $\beta_{\text{max}}$, at all rotation $\alpha$. The second case (Figure S5**B**) shows the need of an extended $\text{DoF}_{\text{misalignment}}$ when the sample, unaligned with the goniometer, travel along the optical axis with the rotation $\alpha$. Theses shifts are the ones retrieved with the alignment algorithm introduced earlier. That is why, to extend depth of field or to cover the necessary range, we choose to bin the pixels of the detector enough (eight times in our case) to reach the required pixel size $\zeta$, according to the $\text{DoF}$ formula for a microscope setup:

|  | $\text{DoF}= \frac{\lambda n_{\text{oil}}}{\text{NA}^{2}}+ \frac{\zeta n_{\text{oil}}}{M \text{NA}}$. | (S6) |
| --- | --- | --- |

On this formula, $\lambda$ is the wavelength of the light used to illuminate the sample, $\text{NA}$ the numerical aperture and $M$ the magnification of the objective.

References

[1] M. Born, E. Wolf, *Principles of optics*, Seventh anniversary edition, 60th anniversary of first edition, 20th anniversary of seventh edition., Cambridge University Press, Cambridge, United Kingdom **2019**.

[2] R. A. Chipman, G. Young, W. S. T. Lam, *Polarized light and optical systems*, Taylor & Francis, CRC Press, Boca Raton **2018**.

[3] E. M. Spiesz, W. Kaminsky, P. K. Zysset, *Journal of Structural Biology* **2011**, *176*, 302.

[4] M. Odstrčil, M. Holler, J. Raabe, M. Guizar-Sicairos, *Opt. Express* **2019**, *27*, 36637.

2. Supporting Information figures


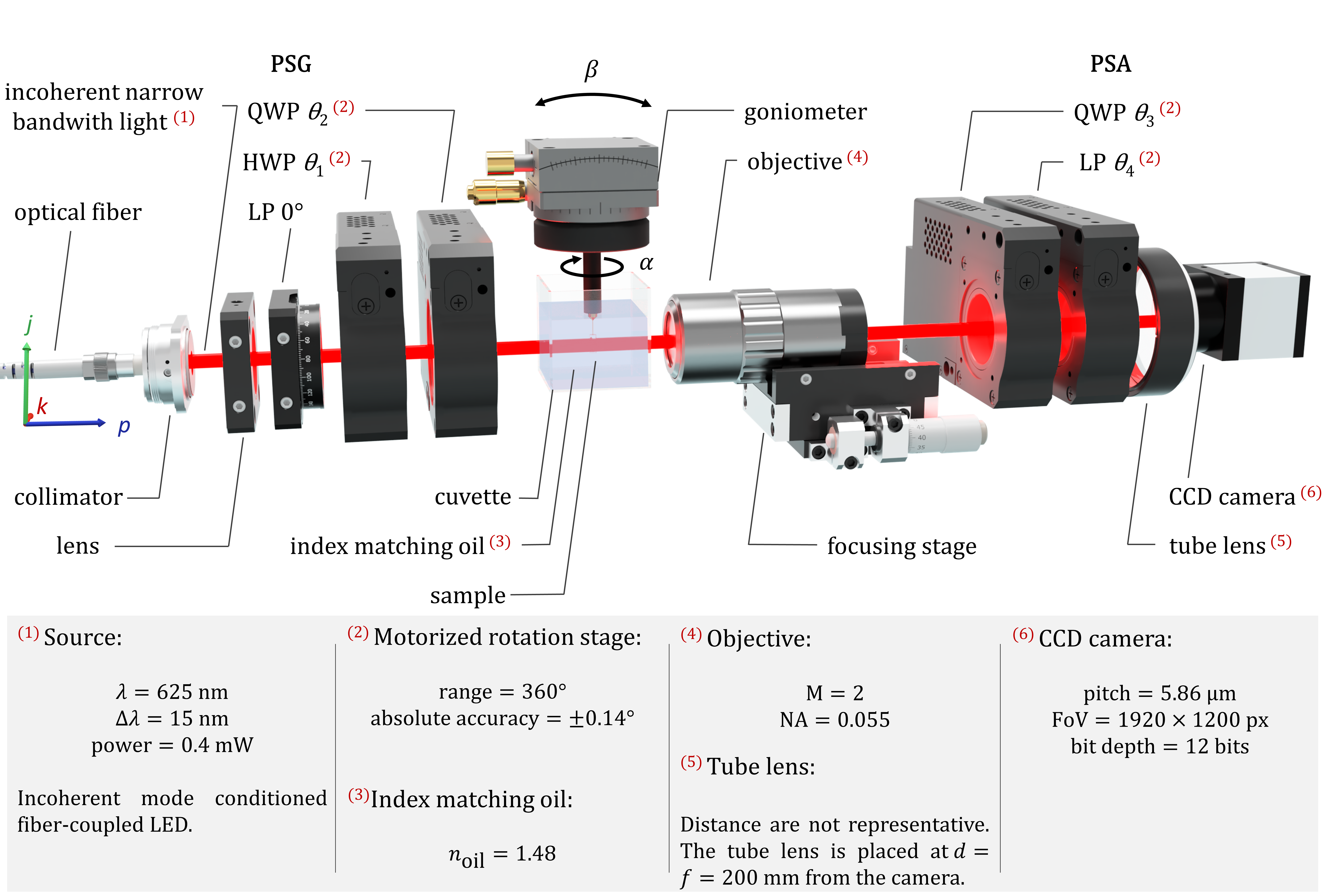


**Figure S1.** Experimental setup. The red light goes from left to right, following the z-axis in the laboratory coordinate system. The polarization state alters through PSG (composed of an LP fixed at $0^{^{\circ}}$, an HWP and a QWP), the sample under investigation, and PSA (composed of a QWP and an LP).


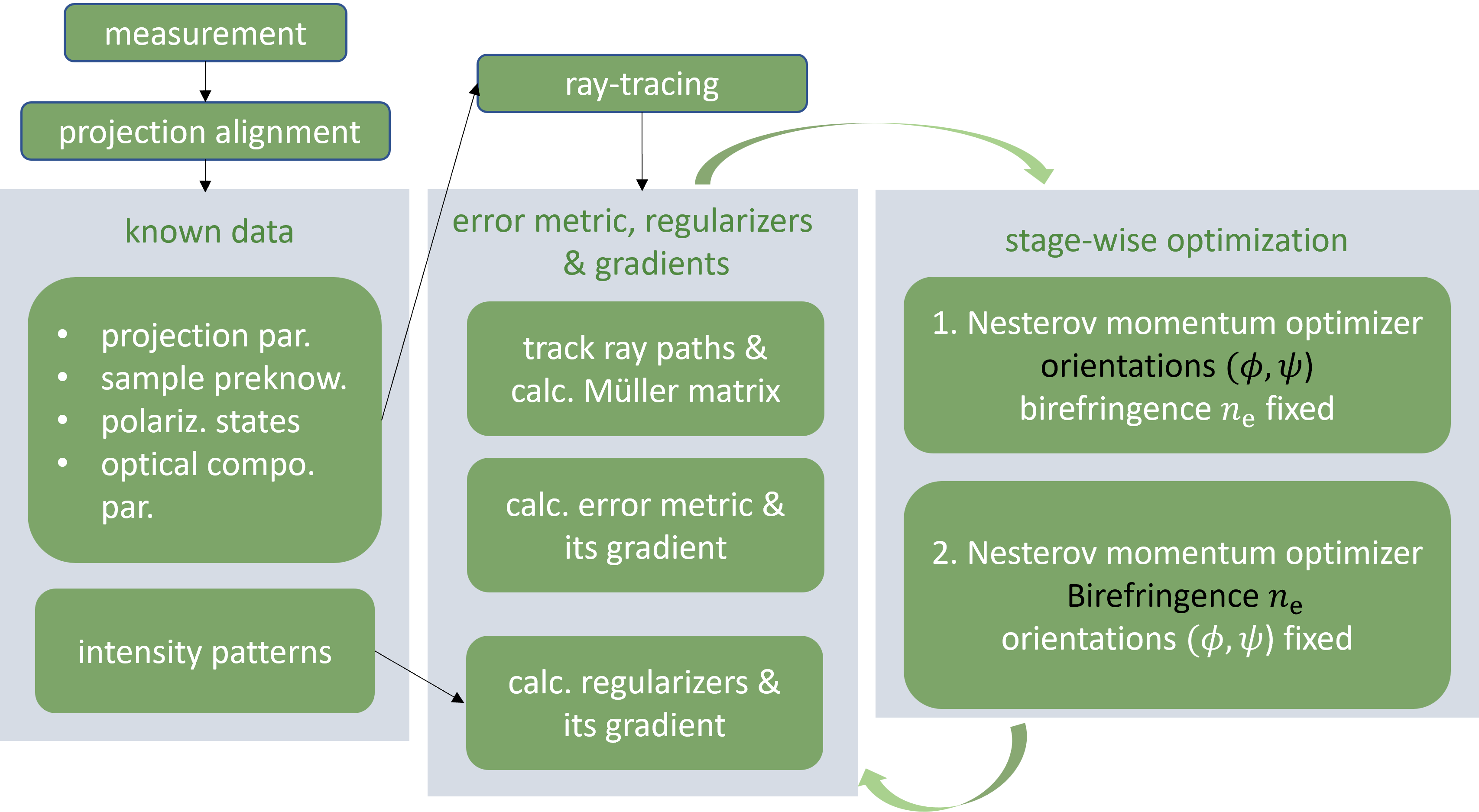


**Figure S2.** Reconstruction framework. The parameters of ($\phi,\psi$) and $n_{e}$ are alternatively optimised in the reconstruction, using the Nesterov accelerated gradient algorithms.


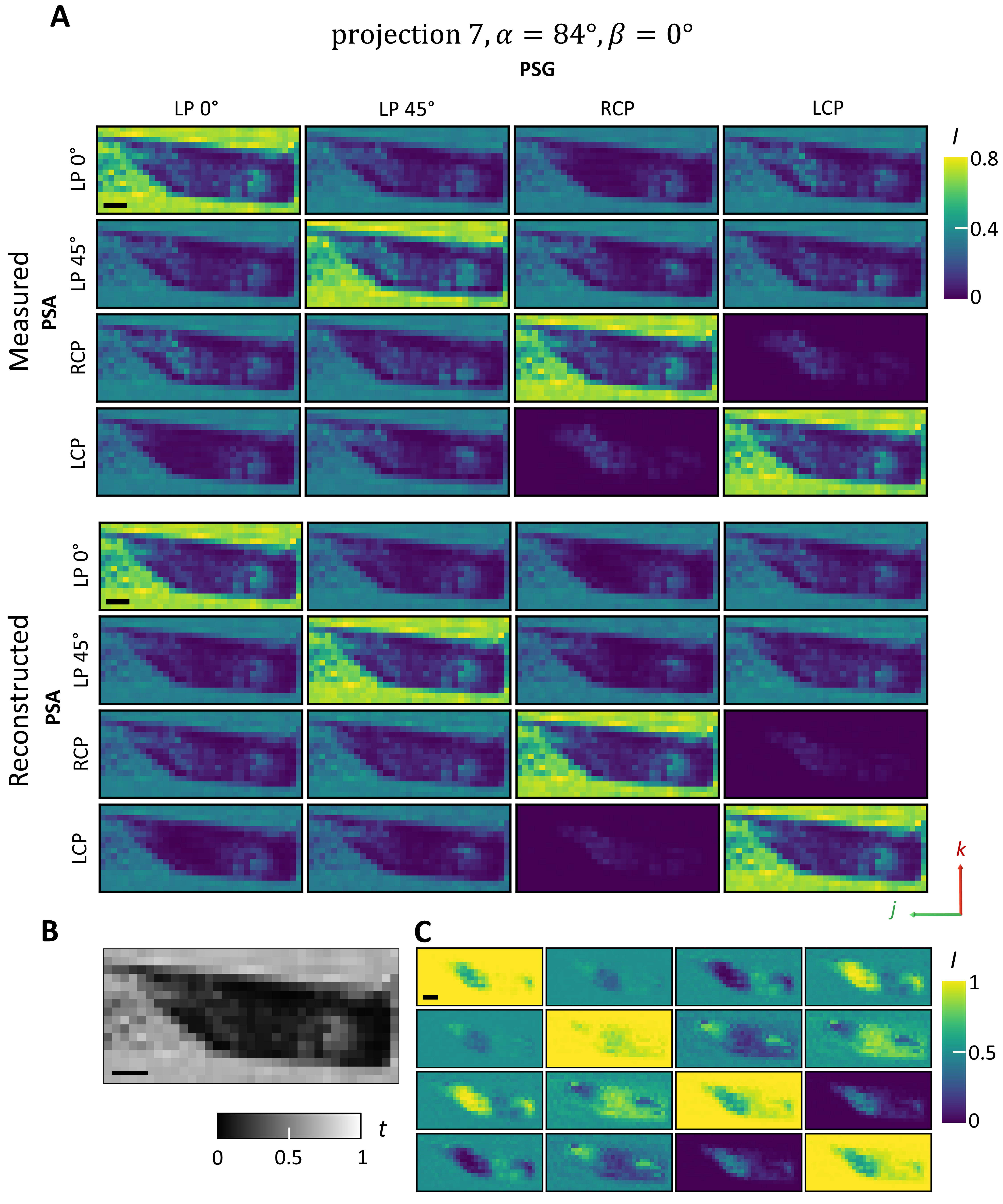


Figure S3. Projections of trabecular bone. (A) The full set of measured intensities $\boldsymbol{I}$ for Projection 7, $\left( \boldsymbol{\alpha,\beta} \right)\boldsymbol{=(}\boldsymbol{84}^{\boldsymbol{^{\circ}}}\boldsymbol{,}\boldsymbol{0}^{\boldsymbol{^{\circ}}}\boldsymbol{)}$, of the measurement (upper) and reconstruction (lower). (B) Extracted transmittance *t*, from the measured data. (C) Intensities reconstructed removing the transmittance *t*. The black bars in B and C indicate a scale of $\boldsymbol{100}\text{μm}$.


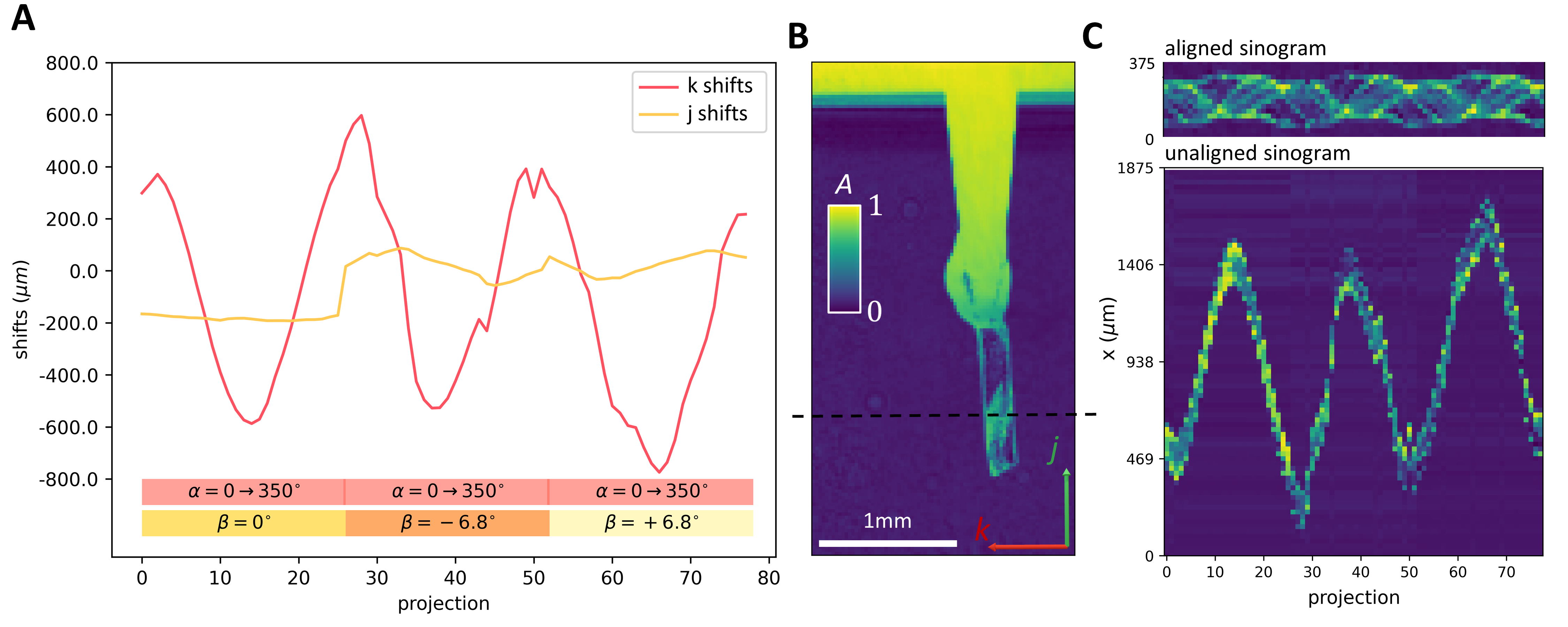


Figure S4. Alignment of the tomographic intensity images. (A) Shifts in the $\boldsymbol{x}$ (red) and $\boldsymbol{y}$ (yellow) directions of the projections calculated by the alignment algorithm based on the 2D absorption maps. (B) Example of an absorption map *A*, from the first projection $\left( \boldsymbol{\alpha,\beta} \right)\boldsymbol{=(}\boldsymbol{84}^{\boldsymbol{^{\circ}}}\boldsymbol{,}\boldsymbol{0}^{\boldsymbol{^{\circ}}}\boldsymbol{)}$. (C) Aligned and unaligned sinogram, cut in the center of sample (see the black dashed line in B).


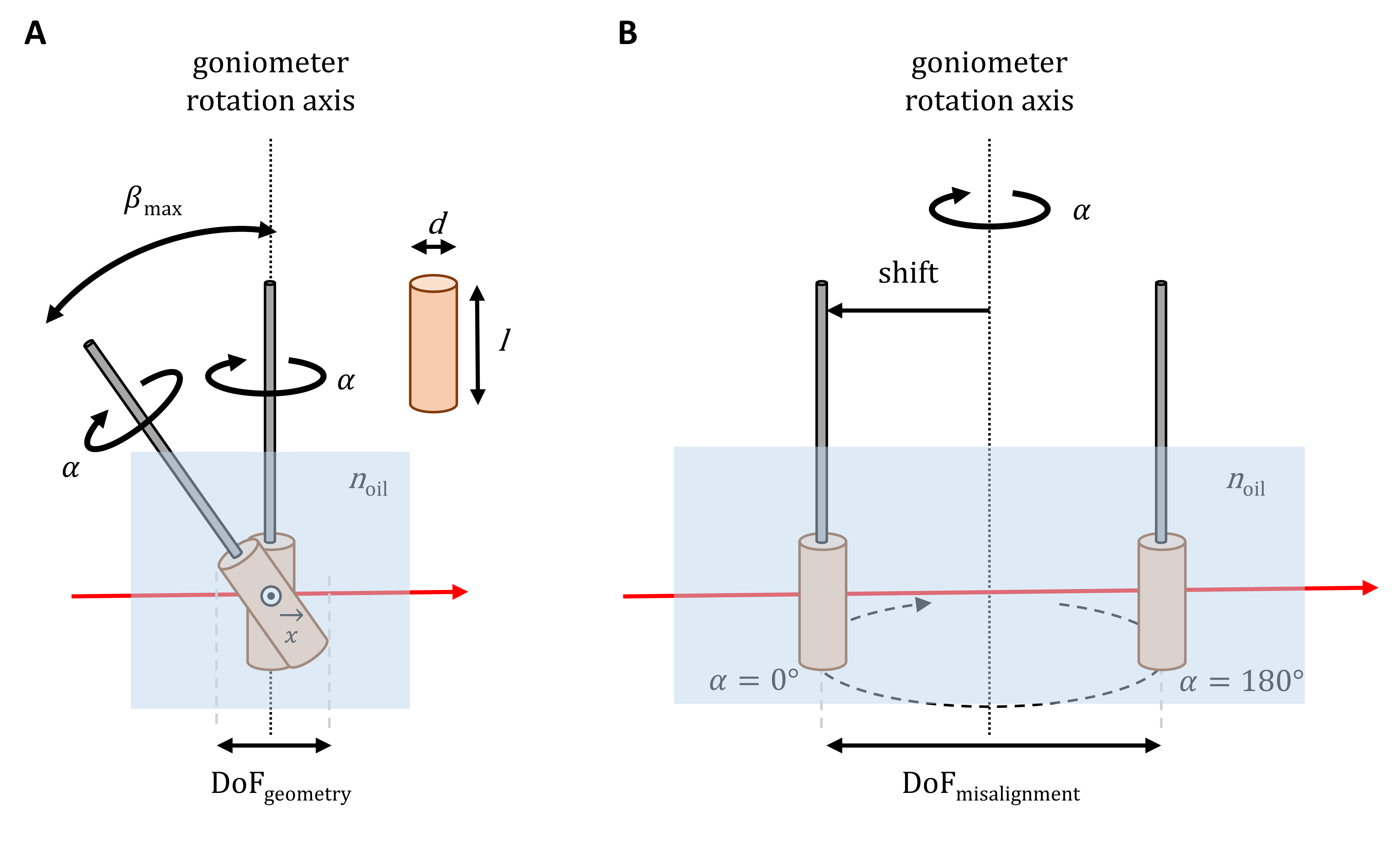


**Figure S5**. Depth of Field in tomography with unaligned sample. (**A**) Depth of Field $\text{DoF}_{\text{geometry}}$ needed for the whole sample to be in focus at all rotation $\alpha$ and tilt $\beta$ up to the maximum tilt $\beta_{\text{max}}$. This depth of field is depending as well refractive index of the medium $n_{\text{oil}}$ on the sample length$l$ and diameter $d$. (**B**) Depth of Field $\text{DoF}_{\text{misalignment}}$ needed for the whole sample to be in focus at all rotation $\alpha$ when the sample is misaligned with the goniometer and translate along the optical axis with the rotation $\alpha$.

3. Legend for Supporting Information movies


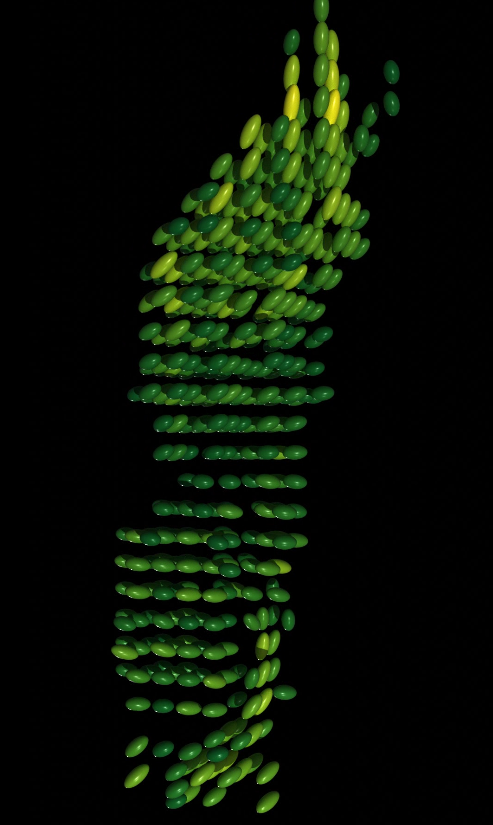


Movie S1. Reconstructed 3D birefringence tomogram of trabecular bone by TMPM. The rotational view, about $\boldsymbol{y}$ axis (see axis definition in Figure 3 of the main text), of the reconstructed 3D index-ellipsoid in each voxel of the bone sample as in Figure 3 and 4 of the main text.
